# Multiparametric super-resolution optoacoustic imaging with ICG-tagged microbubbles

**DOI:** 10.1016/j.mtbio.2025.101865

**Published:** 2025-05-12

**Authors:** Daniil Nozdriukhin, Eva Remlova, Lin Tang, Shuxin Lyu, Gabriella Egri, Ana Torres, Anxo Vidal, Lars Dähne, Daniel Razansky, Xosé Luís Deán-Ben

**Affiliations:** a Institute for Biomedical Engineering and Institute of Pharmacology and Toxicology, Faculty of Medicine, University of Zürich, Switzerland; b Institute for Biomedical Engineering, Department of Information Technology and Electrical Engineering, ETH Zürich, Switzerland; c Departement of Information Technology and Electrical Engineering, ETH Zürich and Max Planck ETH Center for Learning Systems, Switzerland; d Institute of Medical Technology, Shanxi Medical University, Taiyuan, China; e Surflay Nanotec GmbH, Berlin, Germany; f Experimental Biomedicine Centre (CEBEGA), University of Santiago de Compostela, Spain; g Center for Research in Molecular Medicine and Chronic Diseases (CiMUS), Health Research Institute of Santiago de Compostela (IDIS), University of Santiago de Compostela, Santiago de Compostela, Spain

## Abstract

Localization optoacoustic tomography (LOT) has recently emerged as an adept imaging approach for super-resolution functional microangiography at depths out of reach for conventional optical microscopy methods. LOT enables simultaneous measurements of microvascular morphology, oxygen saturation, and blood flow velocity via rapid tracking of highly-absorbing microparticulate agents. These distinctive multiparametric imaging capabilities present a remarkable clinical diagnostic potential for a wide spectrum of conditions associated with microcirculatory alterations. However, wider adoption of LOT is impeded by the lack of contrast materials verified for safe intravenous administration in humans. In this work, we developed a new formulation of polyvinyl alcohol (PVA) microbubbles coated in a layer-by-layer fashion with a sufficiently large amount of indocyanine green (ICG) molecules to enable per-particle detection amid the strong background light absorption of blood. LOT imaging of the murine brain could be achieved with no acute toxicity effects observed. Considering that both microbubbles and ICG are routinely administered in clinical ultrasound and fluorescence angiography procedures, it is anticipated that ICG-coated microbubbles will facilitate the clinical translation of LOT.

## Graphical abstract

**Figure:**
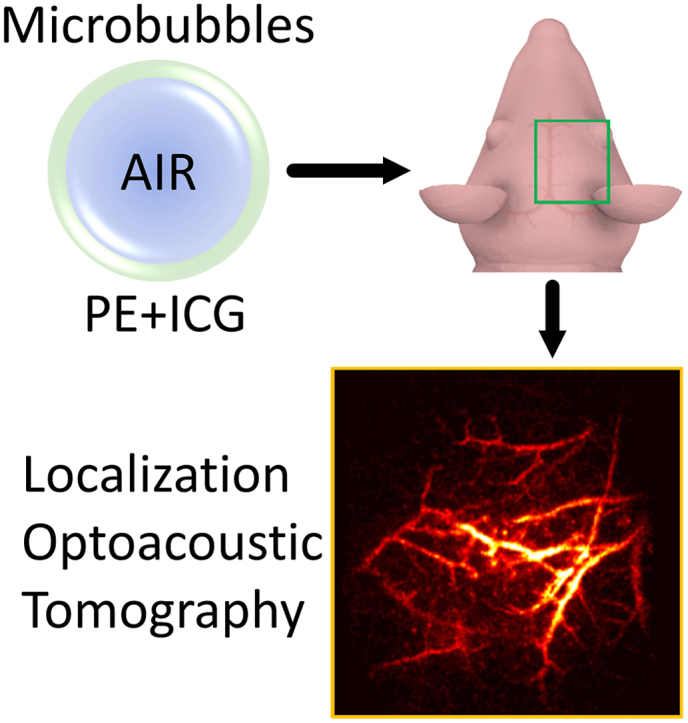

## 1 Introduction

The 21st century has seen a paradigm shift in biomedical imaging with the inception of super-resolution microscopy approaches that elegantly overcome the diffraction-limited resolving capacity imposed by the interrogating wave field [[1], [2], [3], [4], [5], [6], [7]]. A major family of super-resolution methods capitalizes on the fact that the position of isolated sources (particles) in wide field images can be measured with theoretically infinite precision, practically limited by random noise [[8], [9], [10], [11]]. Image formation can then be achieved via superposition of a sufficiently large number of localized particles, allowing for the identification of structures unresolvable with conventional imaging techniques [12].

Localization-based imaging requires hardware and contrast substances enabling per-particle detection in rapidly acquired time-lapse images, along with a strategy to isolate such constitutive particles in an image sequence [13]. The ability to detect fluorescence emission from single molecules laid the groundwork for photoactivated localization microscopy (PALM) imaging of specimens tagged with photoactivatable fluorophores, where localization of activated fluorophores was ensured by randomly switching a sparse subset of these from non-fluorescent to fluorescent states [14]. PALM greatly impacted life sciences by overcoming the optical diffraction limit and enabling the observation of biological processes at the nanoscale [15], whilst ongoing research efforts are further pushing the boundaries of super-resolution optical microscopy [16,17]. Ultrasound (US) imaging has also exploited the same principle to break through the acoustic diffraction barrier deep within biological tissues. So-called ultrasound localization microscopy (ULM), first implemented with conventional B-mode US [18], evolved to capitalize on ultrafast scanners for rapid tracking of intravenously administered microbubbles to reconstruct microvascular structures with capillary-level resolution [[19], [20], [21]]. The sparse distribution of microbubbles facilitates their individual localization, with high-resolution vessel mapping achieved with their accumulated positions as they flow in the bloodstream. Tracking of microbubbles also allows for the assessment of blood flow velocity, leading to a more detailed characterization of the microcirculation [22,23]. A particularly important advantage of microbubble-assisted ULM is its immediate clinical utility as microbubbles are FDA-approved contrast agents regularly injected in patients to enhance the angiographic and blood flow imaging capacity of pulse-echo US. Indeed, ULM has been used to image different parts of the human body such as brain, kidneys, or liver [[24], [25], [26]].

Optoacoustic (OA, photoacoustic) tomography has also gained recognition as a powerful angiographic imaging technique [27,28]. The high and spectrally-distinctive absorption of hemoglobin in oxygenated and deoxygenated forms allows for label-free visualization of microvascular networks and oxygen saturation mapping. This unique functional imaging capacity has been exploited in preclinical studies in oncology [[29], [30], [31], [32]], neuroscience [[33], [34], [35], [36]], cardiovascular biology [[37], [38], [39], [40], [41]], and other fields [[42], [43], [44], [45]]. Recently, multiple clinical trials have further demonstrated the potential of OA as a diagnostic tool [[46], [47], [48], [49]], and commercial systems have been certified for clinical use. This anticipates a growing dissemination of the OA technology in the clinical setting. Much like for pulse-echo US, the resolution of OA tomography is fundamentally limited by acoustic diffraction [50]. Conversely, volumetric OA images can be rendered with single laser pulses at rates of hundreds to thousands frames per second without the need to compound multiple images [[51], [52], [53]]. This results in an effective integration time of a few nanoseconds, which facilitates localization of point sources with high precision. However, implementation of localization-based imaging in OA tomography has been challenged by the relatively weak signals generated by small absorbers in the presence of the strong background absorption by blood. Recently, localization optoacoustic tomography (LOT) has been achieved via intravenous injection of droplets exhibiting three to four orders of magnitude higher absorption than red blood cells (RBCs) [54]. However, bio-safety concerns related to micro-droplet formulations substantially hamper their potential clinical translation. Herein, we introduce a new type of polyvinyl alcohol (PVA) microbubbles coated with indocyanine green (ICG). The main goal is to create a contrast agent composed of clinically safe materials to potentially unlock the capabilities of LOT for clinical diagnostics. Additionally, ICG-tagged microbubbles can serve as contrast agents for other modalities, including US or fluorescence imaging, thus may facilitate the development of powerful multi-modal imaging approaches.

## 2 Results

### 2.1 Stable ICG-coated PVA microbubbles

**Fig. 1:**
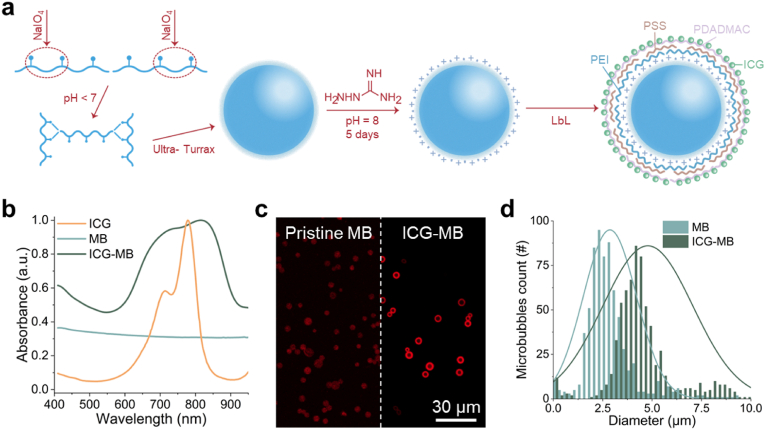
**Synthesis of indocyanine green (ICG)-coated poly(vinyl alcohol) (PVA) microbubbles.** (a) Steps for microbubble synthesis and layer-by-layer (LBL) coating with ICG. PEI - poly(ethylene imine), PSS - poly(styrene sulfonate), PDADMAC - poly(diallyl-dimethylammonium chloride). (b) Normalized absorbance of pristine microbubbles, ICG-coated microbubbles and pure ICG. (c) Confocal microscopy images of pristine (left) and ICG-coated (right) microbubbles. (d) Size distributions of pristine (2.79 ± 1.23 μm) and ICG-coated (4.79 ± 2.26 μm) microbubbles. (For interpretation of the references to color in this figure legend, the reader is referred to the Web version of this article.)

Stable air-filled PVA microbubbles were produced through a cross-linking reaction at the air-water interface, with the average bubble size being established by the reaction temperature. Following the introduction and stabilization of a positive charge on the surface of the microbubbles, ICG molecules were added using a layer-by-layer coating procedure (Fig. 1a, see methods for details). Optical spectroscopy distinctly confirmed the presence of ICG in the coated bubbles, characterized by an absorption peak at ∼800 nm (Fig. 1b). Single-bubble OA sensitivity was demonstrated by imaging the flow of microbubbles in a polyethylene tubing phantom (Video S1). The characteristic absorption spectrum of the bubbles may serve to amplify the target per-particle signals via multi-spectral (multi-wavelength) data acquisition, which is however challenged by the fast motion of the microbubbles in blood.

**Fig. 2:**
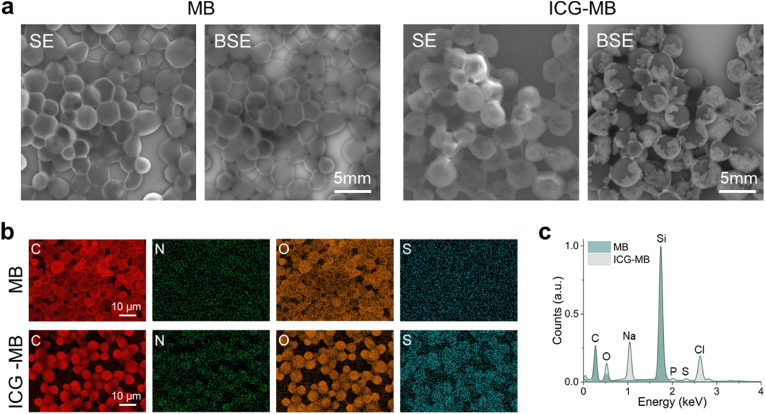
**Morphology and composition of the microbubble shell.** (a) Scanning electron microscopy (SEM) images of pristine (left) and ICG-coated (right) microbubbles. Secondary electron (SE) and back-scattered electron (BSE) modes are shown. (b) Energy-dispersive X-ray spectroscopy (EDS) elemental mapping of carbon (C), nitrogen (N), oxygen (O), and sulfur (S) for pristine (top) and ICG-coated (bottom) microbubbles. (c) EDS spectra for pristine and ICG-coated microbubbles.

Confocal microscopy images also clearly indicated an increased fluorescent signal of the ICG-coated microbubbles with respect to the pristine ones associated with the presence of the dye (Fig. 1c). The ICG content per bubble was estimated to be ∼113.5 pg/bubble based on the ICG calibration curve and the absorption of shockwave-destroyed microbubbles suspension at 640 nm (Fig. S1). The size distributions for pristine (2.79 ± 1.23 μm) and ICG-coated (4.79 ± 2.26 μm) microbubbles, measured with bright-field microscopy images (see methods for details), were also slightly different arguably due to the shell loading the ICG molecules (Fig. 1d). Designed with a diameter smaller than RBCs, the synthesized ICG-coated PVA bubbles are expected to seamlessly circulate through microvascular networks without inducing capillary arrest. They are also shown to exhibit higher monodispersity compared to commercial microbubbles commonly used as clinical US contrast agents [55]. This is of importance to achieve consistent imaging results as well as for a more predictable biodistribution and clearance. Scanning electron microscopy (SEM) images of the microbubbles also revealed clear differences between pristine and ICG-coated bubbles (Fig. 2). Specifically, a shell corresponding to the multi-layered polyelectrolyte structure embedding the ICG molecules was observed in the coated bubbles. This is more prominent in back-scattered electron (BSE) images as contrast is provided by heavier elements such as sulfur in ICG (Fig. 2a). On the contrary, both BSE and secondary electron (SE) SEM images indicated a smooth surface of the pristine bubbles. The shell is expected to increase the rigidity of the bubbles. Rigid microbubbles are less likely to result in dissolution of the gas core and clearance through the lungs, which leads to a longer circulation time in the bloodstream. The composition of the coating was also verified with energy-dispersive X-ray spectroscopy (EDS) elemental analysis guided with the SEM images (Fig. 2b). The presence of the sulfur peak corresponding to ICG was also observed in the EDS spectrum of the coated bubbles (Fig. 2c).

### 2.2 Cell viability and biosafety

**Fig. 3:**
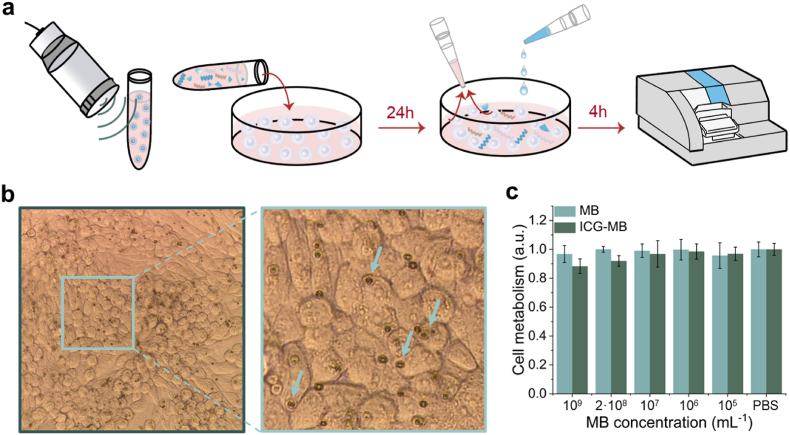
**Cytotoxic effects of indocyanine green (ICG)-coated microbubbles.** (a) Steps of the cell viability assay. From left to right – 1) microbubble disruption with ultrashort ultrasound pulses, 2) feeding of the Chinese hamster ovary (CHO) cell culture with microbubble debris, 3) addition of alamarBlue solution to the cell culture and subsequent sampling, 4) measurement of fluorescent signals. (b) Bright field optical image of the cells prior to addition of alamarBlue solution. Internalized particles are indicated with arrows. (c) Results of the alamarBlue cell viability assay for cells incubated with different concentrations of ICG-coated microbubbles. (For interpretation of the references to color in this figure legend, the reader is referred to the Web version of this article.)

The biocompatibility and potential toxic effects of the ICG-coated PVA microbubbles were assessed with two different approaches. First, a cell viability study was conducted on Chinese hamster ovary (CHO) cultured cells as they represent normal non-tumorous epithelial-like mammalian tissue and widely used in toxicology research and industrial drug testing due to their well-characterized properties (Fig. 3). Considering that PVA is a synthetic biocompatible polymer and that ICG is approved for clinical use, no harmful effects are expected from the main materials. However, other shell components could potentially lead to toxic effects. Thereby, the microbubbles were ruptured by exposing them to high-intensity ultrashort US pulses prior to exposing the CHO cells to the resulting debris (Fig. 3a, see methods for details). This also ensured that the particles were in touch with the cells, as otherwise, they tended to float in the culture medium. Indeed, internalization of particles by the cells was observed in bright field optical images (Fig. 3b). Cell viability was evaluated using an alamarBlue assay after 24 h of exposure to various concentrations of microbubble debris (5 × 10^5^ to 5 × 10^8^ microbubbles per mL). This test revealed that CHO cells maintained viability, with slightly increased alamarBlue fluorescent signals observed in some cases suggesting that they can increase metabolism to digest the internalized substances (Fig. 3c, Fig. S2).

**Fig. 4:**
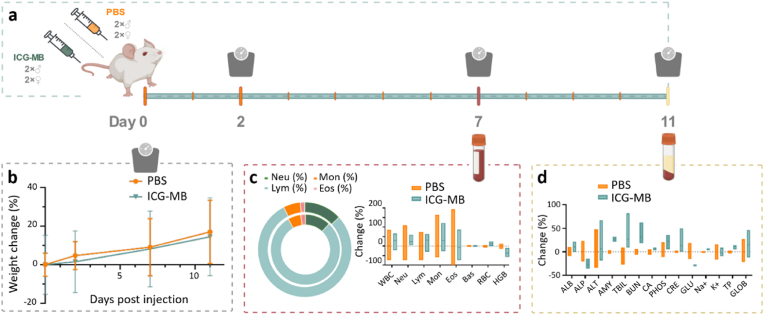
***In vivo* biosafety of indocyanine green (ICG)-coated microbubbles.** (a) Timeline of the biosafety study for two groups of mice (n = 4) injected with ICG-coated microbubbles and phosphate buffered saline (PBS). (b) Mouse weight monitoring in the two groups of mice. (c) Blood hematology taken on day 7 post-injection. WBC – white blood cells: Neu – neutrophils, Lym – lymphocytes, Mon – monocytes, Eos – eosinophils, Bas – basophils, RBC – red blood cells, HGB – hemoglobin. (d) Blood biochemistry taken on day 11 post-injection. ALB – albumin, ALP - alkaline phosphatase, ALT - alanine transaminase, AMY – amylase, TBIL - total bilirubin, BUN – blood urea nitrogen, CA – calcium, PHOS – phosphates, CRE – creatinine, GLU – glucose, Na + - sodium, K + - potassium, TP – total protein, GLOB – globulin. Percentual variations with respect to the average values for the control group are shown. (For interpretation of the references to color in this figure legend, the reader is referred to the Web version of this article.)

*In vivo* biosafety was further studied on Swiss mice as they are a commonly used, non-immunosuppressed model organism suitable for such evaluations. For this, two groups of mice (n = 4, 2 males and 2 females per group) received an intravenous injection of a suspension of ICG-coated microbubbles (100 μL of 5 × 10^8^ bubbles per mL) or phosphate buffered saline (PBS; 100 μL, control group) and blood was extracted 7 days post-injection after longitudinal monitoring and scoring of the mice for behavior and physical appearance (Fig. 4). No weight loss was observed in any of the mice (Fig. 4b), with all scoring parameters remaining as expected for healthy mice. Hematology analysis revealed that the immune cell counts were similar between the nanoparticle-treated and control groups, indicating no induction of acute inflammation (Fig. 4c). Blood biochemical analysis further showed no ramp up in key health indicators such as alkaline phosphatase, alanine transaminase, and blood urea nitrogen compared to the control group (Fig. 4d, Fig. S3 and S4), signifying no liver and kidney damage, or red blood cells lysis. The biodistribution assay demonstrated the main accumulation of the ICG-MB in liver 60 min post-injection (Fig. S5).

### 2.3 Functional imaging of unresolvable vessels

**Fig. 5:**
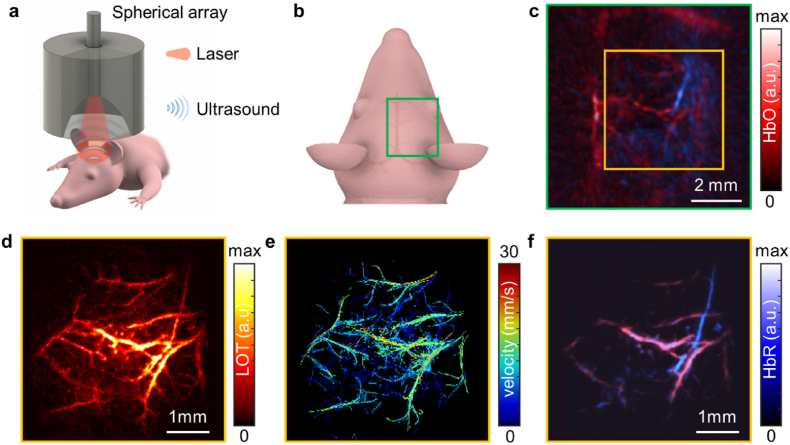
**Super-resolution optoacoustic (OA) imaging of the murine cortex with indocyanine green (ICG)-coated microbubbles**. (a) Lay-out of the three-dimensional OA imaging system used in the experiments. (b) Imaged area in the mouse brain. (c) Maximum intensity projection (MIP) of the reconstructed OA image unmixed for oxygenated (red) and deoxygenated (blue) hemoglobin. (d) Localization optoacoustic tomography (LOT) image for the area indicated in panel (c). (e) Blood flow velocity map estimated via microbubble tracking. (f) Biodistributions of oxygenated and deoxygenated hemoglobin in microvascular structures resolved with LOT. (For interpretation of the references to color in this figure legend, the reader is referred to the Web version of this article.)

OA imaging provides a unique capability to spectrally differentiate specific absorbing molecules at depths beyond the range of optical microscopy. For this, sequential tissue excitation at multiple wavelengths is performed, followed by multi-spectral (multi-wavelength) unmixing of the collected data. This is of particular relevance to quantify oxygen saturation in optoacoustically-resolvable vascular networks, thus rendering otherwise-unattainable functional information [56,57]. Multi-spectral OA imaging of the nude mouse brain vasculature was performed with a custom-made spherical array featuring a central aperture for guiding the output beam of an optical parametric oscillator (OPO)-laser tunable in the NIR range (Fig. 5a, see methods for details). The mouse strain was chosen due to the hairless nature and lack of melanin, which simplifies animal preparation. This selection also helps minimize the total number of animals used, as it prevents the exclusion of subjects with excessive melanin patches on the scalp that could interfere with imaging. The region of interest was chosen to cover an area between the central superior sagittal sinus and the middle cerebral artery (Fig. 5b). The reconstructed OA image, unmixed for oxygenated and deoxygenated hemoglobin, covered major vessels in this area within the ∼150–250 μm resolution range provided with the system (Fig. 5c). Multi-spectral unmixing the expected dominance of oxygenated hemoglobin throughout the brain as well as the presence of deoxygenated hemoglobin in some regions. Following acquisition of multi-spectral data, a sequence of OA images at 800 nm excitation wavelength and 100 frames per second was acquired during injection of a 100 μL bolus of ICG-coated microbubbles (see methods for details). Visualization of individual bubbles flowing in blood was possible after singular value decomposition (SVD)-filtering of the acquired sequence of frames (Video S2). It is shown that such dots are mainly visible in the central area of the OA field of view, suggesting a non-linear dependence of the OA responses from individual microbubbles with fluence. A LOT image was reconstructed by considering the localized positions of ∼100000 individual bubbles (Fig. 5d, Fig. S6). Microvascular structures not resolvable in the OA image were clearly visible in the LOT image. The limited depth covered with LOT relative to OA also suggests a non-linear response with fluence. Tracking of particles further enables calculating a blood flow velocity map providing functional angiographic information (Fig. 5e, see methods for details). More importantly, the LOT image enabled building a mask in which oxygen saturation was calculated. The estimated oxygenation values are shown to match the super-resolved vessels (Fig. 5f), indicating that LOT facilitates oxygen saturation mapping within vascular networks not resolved in the original diffraction-limited OA images. With this, LOT achieves a unique multi-parametric imaging performance including super-resolution angiography, blood flow velocity mapping, and oxygen saturation estimation. The depth resolving capacity of LOT is better visualized in rotating views of the LOT image, the velocity map, and the superimposed oxygenated and deoxygenated components (Video S3).

## 3 Discussion and conclusions

Emerging imaging technologies enabling improved characterization of microvascular morphology and function can make a substantial impact in clinical diagnostics [58]. Oxygen and nutrients are primarily exchanged around vessels smaller than 100 μm that support cellular function throughout the body. This is compromised in multiple diseases affecting the microcirculation, including cancer, diabetes, peripheral vascular disease, arthritis, or atherosclerosis, to name a few examples [59,60]. The ability to accurately visualize and assess the induced circulatory changes is expected to facilitate early detection, precise monitoring, and tailored therapeutic strategies, thus can potentially lead to a significant step forward in the clinical management of complex conditions. A range of optical techniques can readily achieve microscopic imaging of shallow microvasculature structures [61,62]. However, medical imaging techniques needed to access most subcutaneous areas typically lack the resolution needed for imaging at this fine scale. Innovative approaches to increase the resolution of OA and US imaging have led to previously unavailable capabilities for microvascular imaging. OA mesoscopy systems can resolve microvascular networks with dimensions as small as 10–30 μm for depths up to 2–3 mm within biological tissues [63,64]. On the other hand, the resolution of ULM is ultimately limited by the signal intensity from individual microbubbles and can theoretically reach the capillary level (∼5 μm) at larger depths [65].

LOT synergistically combines the advantages of OA and super-resolution angiography. A major strength of OA lies in its capability to provide functional angiographic contrast in three dimensions [54]. Specifically, spectral differences in the optical absorption of oxygenated and deoxygenated hemoglobin are exploited in multi-spectral (multi-wavelength) OA imaging to offer detailed insights into oxygen saturation levels within the vasculature, a critical parameter for assessing tissue viability and metabolic status [66]. OA further provides molecular information by resolving targeted dyes and nanoparticles or light-absorbing proteins [67]. Also important is the fact that higher signal-to-noise (SNR) can be achieved for superficial particles in LOT relative to ULM, as shown in the original manuscript demonstrating the LOT applicability *in vivo* [54]. With the development of ICG-tagged microbubbles, we anticipate further facilitating the clinical translation of LOT. Alternatively, ULM excels in its ability to visualize microvascular networks with spatial resolution beyond the acoustic diffraction limit and additionally provides a unique capability to quantify blood flow velocity [68]. This is poised to shed new light onto microvascular function and contribute to a better understanding of microcirculatory dynamics. LOT simultaneously provides 1) three-dimensional angiographic imaging beyond the acoustic diffraction barrier, 2) blood flow velocity quantification, and 3) oxygen saturation mapping [54]. Thereby, LOT can emerge as an unprecedented tool in biomedical imaging providing multi-parametric characterization of tissue physiology and enabling a better understanding of diseases characterized by alterations in microvascular integrity and function. Relevant disease models may include subcutaneous or orthotopic tumors, neurodegenerative diseases, or metabolic disorders [34,42,69]. It is also important to note that ICG-tagged microbubbles can be used for both OA and US, potentially providing hybrid super-resolution angiography capitalizing on the advantages of both modalities. Phase-change nano-droplets activated with light or sound can also potentially be used for hybrid imaging [70,71]. Light activation was exploited to localize the resulting bubbles using US [72], while nanodroplets have also been successfully utilized for ULM [21,73,74].

The development of LOT as a super-resolution OA imaging modality has been hampered by the lack or particulate contrast agents capable of being individually detected as they flow thorough the bloodstream along with highly-concentrated red blood cells strongly absorbing light. Recently, microdroplets [54], microcapsules [75], solid-core microparticles [76,77], and petal-shaped microflowers [78] smaller than red blood cells could be individually detected and tracked *in vivo* following intravenous (tail vein) injection in mice. This empowers LOT with a unique versatility for microparticle tracking, which not only facilitates achieving super-resolution angiography but may additionally enable other new applications e.g. in drug delivery or biosensing [79]. However, none of the aforementioned formulations is currently used in the clinical setting. In contrast, microbubbles and ICG are FDA-approved agents regularly used for achieving enhanced angiographic contrast [80]. The ICG-coated PVA microbubbles introduced in this work are then poised to facilitate regulatory approval and enable a clinical translation of LOT. The suggested layer-by-layer approach for ICG labelling is also potentially applicable to other types of microbubbles based on different shell materials and encapsulated gases available, which may further facilitate clinical acceptance. PVA microbubbles feature important advantages, including greater mechanical stability and longer circulation due to their rigid, cross-linked shell. Their polymer structure allows easy functionalization for targeting or drug delivery, unlike more complex phospholipid systems. PVA microbubbles also resist degradation during storage and in biological environments, outperforming lipid-based alternatives. However, lipid-based microbubbles remain the gold standard in clinical applications due to their excellent biocompatibility and well-established safety profile, and the feasibility of ICG labelling has also been demonstrated [81].

In conclusion, LOT assisted with ICG-based microbubbles may lead to a significant leap in medical imaging, merging super-resolution capabilities with functional microangiography to delineate microvascular dynamics in unprecedented detail. The developed ICG-coated PVA microbubbles align with contrast agents used in clinical practice, thus offering high translational potential. The demonstrated safety in murine models defines a pivotal milestone toward the applicability of LOT in human diagnostics, which is anticipated to establish a new paradigm in the visualization and understanding of diseases marked by vascular alterations.

## 4 Materials and methods

### 4.1 Materials

Poly(vinyl alcohol) (PVA, 70 kDa), sodium periodate (NaIO_4_), indocyanine green (ICG), aminoguanidine (AG), poly(ethylene imine) (PEI, 25 kDa), poly(styrene sulfonate) (PSS, 70 kDa), poly(diallyl-dimethylammonium chloride) (PDADMAC, 200–400 kDa), Hepes buffer (pH 8) were purchased from Sigma-Aldrich. Double-deionized water (specific resistivity 18.2 MΩ cm) produced by MilliQ System was used to make all the solutions. Fetal Bovine Serum (FBS), Dulbecco's Modified Eagle Medium (DMEM) and alamarBlue cell viability assay were purchased from Thermo Fisher Scientific. Chinese Hamster Ovarian (CHO) cells were obtained from CLS Cell Lines Service GmbH.

### 4.2 Synthesis of microbubbles

PVA microbubbles were prepared based on a protocol resulting in a cross-linking reaction at the air-water interface [82]. As the first step, the vicinal hydroxyl groups of the PVA were cleaved and oxidized using sodium periodate, resulting in the chains of 50 monomers in length, followed by cross-linking through ketal formation under acidic conditions. The microbubbles, approximately 3 μm in size, were produced by dispersing the atmospheric air with a cell homogenizer (Ultraturrax T-25, IKA) at high rpm into the obtained solution of the processed branched polyketal PVA.

A positive surface charge was imparted by covalently coupling AG to the remaining aldehyde groups in a 5 g/L solution in 0.1 M Hepes buffer at pH 8 for five days at room temperature. The surface charge was stabilized by applying 2.5 double layers of PEI and PSS using a layer-by-layer approach [83], followed by a single layer of PDADMAC to maintain the strong positive zeta-potential.

### 4.3 Layer-by-layer ICG coating

ICG added to the aqueous solution (1 mg/mL) was strongly adsorbed to the surface of the microbubbles by capitalizing on the negative charge of the molecule and the fact that it exhibits high affinity towards PDADMAC. This forms the outermost, negatively charged, stable layer of ICG. The obtained ICG-labelled microbubbles were rinsed with DDI water five times in the centrifugation cycle to ensure that no free ICG was left in the supernatant. The fluorescence signal of the microbubbles was studied using confocal microscope (Zeiss LSM800) using 20× objective at 640 nm excitation wavelength and 680 nm longpass emission filter. Both ICG-coated and pristine bubbles diameters were assessed using a brightfield optical microscope (Primostar 3, Zeiss) equipped with a digital camera (Prime BIS Express, Photometrics). The images were thresholded and the diameters of the bubbles were calculated using ImageJ 1.54.

### 4.4 Scanning electron microscopy and EDS

The morphology of the microbubbles and the shell were assessed with SEM. All samples were deposited on a dried p-doped silicon chip (Electron Microscopy Sciences, USA) cleaned consecutively with absolute ethanol and DDI water in an ultrasonic bath for 10 min. The silicon chip was mounted onto a standard SEM stub by using carbon duct tape and the microphotographs were obtained using Hitachi SU5000 microscope. The accelerating voltage was set to 10 kV for both SE and BSE modes. Energy dispersive spectroscopy analysis was carried out using two Ultim Max 100 detectors (Oxford Instruments) mounted at 90° at 10 kV accelerating voltage.

### 4.5 Optical spectroscopy

UV–Vis–NIR spectroscopy measurements were performed using an Infinite M200 + well-plate reader (Tecan, Austria). A 200 μL microbubbles suspension was placed in a 96-well plate (Nunclon Delta, Thermo Fisher Scientific, Denmark) and the absorbance was measured in the wavelength range of 350–900 nm. Data was then processed using Origin 2024 software.

### 4.6 Cell viability

The biocompatibility of the microbubbles was evaluated using an alamarBlue assay conducted on a Chinese hamster ovary (CHO) cell culture. A 200 μL of cell suspension with 8 × 10^3^ cells in DMEM supplemented with 10 % fetal bovine serum (FBS) was seeded into each well of a 96-well plate and cultivated in a humidified incubator with 5 % CO_2_ at 37 °C for 24 h to ensure the cell attachment and proliferation. The microbubbles suspension was exposed to ultrashort US pulses at 0.25 mJ/cm^2^ (Neurolith, Storz Medical) energy level to disrupt their walls therefore reducing their positive buoyancy and facilitating the cellular uptake on the bottom of the 96-well plate. After three rinsing cycles with PBS, the cell culture was fed with 20 μL of deflated microbubbles suspension at different concentrations (5 × 10^5^ to 5 × 10^8^ microbubbles per mL) and PBS as the control. The cells were incubated with the microbubble suspension for an additional 24 h, which is enough for the acute toxicity assessment, but short enough to avoid additional effects of the cell proliferation since the CHO culture has a 20–24 h of the cell doubling time. The cells were rinsed with PBS to remove the microbubbles not being uptaken and supplemented with 20 μl of the alamarBlue solution for 4 h. The supernatant in each well was then collected to a fresh 96-well plate to create a reading replica and the fluorescent signal from each well was measured at 540 nm excitation/590 nm emission wavelengths using Infinity M200+ well-plate reader.

### 4.7 *In vivo* toxicity study

The biosafety of the synthesized microbubbles was assessed in Swiss mice (n = 8, 7 weeks old, 4 males and 4 females) split into experiment (n = 4, 2 males and 2 females) and control (n = 4, 2 males and 2 females) groups injected with a 100 μL bolus of PBS and a suspension of 5 × 10^8^ ICG-coated microbubbles per mL. Mice were scored and weighted on days 2, 7, and 11 following an i.v. injection, and subsequently sacrificed. Hematological analysis of blood samples was performed by using a BC5000-Vet analyzer (Mindray) at day 7 post-injection. Clinical biochemistry assay was performed using a VetScan VS2 analyzer (Zoetis) with blood samples collected after sacrifice the mice at day 11 post-injection. The biosafety study was done following Spanish and European regulations and approved by the Xunta de Galicia.

### 4.8 Multi-spectral optoacoustic imaging

Multispectral optoacoustic tomography (MSOT) of a mouse brain was performed with a custom-made 512-element spherical array transducer with central frequency of 7 MHz [84]. OA excitation was performed with the output light beam of a tunable pulsed optical parametric oscillator (OPO)-based laser (6 ns pulse duration, ∼20 mJ/cm^2^ fluence at the tissue surface, Innolas EVO II, Germany). This was guided to illuminate the sample using a custom-made silica fiber bundle (NA = 0.27, Lightguide, Germany). The acquired signals were averaged 50 times for each of the selected 5 optical wavelength, namely 700, 730, 760, 800, and 850 nm. For imaging, the eyes of the animal were covered with dexpanthenol cream, and the head was fixed in a stereotactic frame to prevent the artefacts from breathing motion. The mouse was placed in a prone position on a heating pad covered with soft tissue to maintain a constant body temperature (37 °C). All experiments were performed following the Swiss Federal Act on Animal Protection and were approved by the Cantonal Veterinary Office Zürich.

### 4.9 Localization optoacoustic tomography

Following multispectral data acquisition, a bolus of 100 μL of the ICG-labelled microbubbles at an approximate concentration of 5 × 10^8^ microbubbles per mL was injected into the tail vein. OA images were captured at 800 nm excitation wavelength and 100 Hz pulse repetition frequency for 3 min (18000 frames). The laser beam was guided through a 5 mm core silica fiber bundle with NA = 0.27 inserted into the transducer array central cavity. Localization optoacoustic tomography (LOT) images were reconstructed from the captured datasets using the methodology outlined elsewhere [54]. Briefly, the raw signals underwent bandpass filtering with cut-off frequencies of 1–7 MHz. Subsequently, a singular-value decomposition (SVD) filter was applied to eliminate tissue clutter and high-frequency noise in subsets of 200 frames. This involved filtering the eigenvectors within the range of 31–130, corresponding to signals emitted by fast-moving microbubbles, using Casorati matrices of subsets sized (493 samples x 512 transducer elements) x 200 frames. A tracking algorithm (simpletracker.m, available on MathWorks ©Jean-Yves Tinevez, 2019, wrapping MATLAB Munkres algorithm implementation of ©Yi Cao 2009) was then employed to estimate microbubble displacement across consecutive frames. A maximal linking distance of 0.5 mm was selected, corresponding to a maximum particle velocity of 50 mm/s, considering the imaging frame rate. The minimum track length of 4 points and gap closing distance of 1 was used, while the image resolution was 50 μm/pixel.

### 4.10 Multispectral unmixing of oxygenated and deoxygenated hemoglobin

The biodistributions of oxygenated and deoxygenated hemoglobin were assessed by normalizing single-wavelength images with optical fluence, estimated using an ink phantom measured at the same position. This was followed by standard spectral fitting of images at various wavelengths to the absorption spectra of the two hemoglobin forms. Given that the murine cortex is a shallow structure, the spectral coloring effects due to wavelength-dependent light attenuation are not expected to significantly affect the measurements. A mask combining the LOT image and the vesselness filtered LOT image was considered to estimate the oxygenated and deoxygenated content of the super-resolved image.

## CRediT authorship contribution statement

**Daniil Nozdriukhin:** Writing – review & editing, Writing – original draft, Investigation, Formal analysis, Data curation. **Eva Remlova:** Writing – original draft, Visualization, Formal analysis, Data curation. **Lin Tang:** Writing – review & editing, Visualization, Data curation. **Shuxin Lyu:** Writing – review & editing, Investigation, Data curation. **Gabriella Egri:** Methodology, Investigation, Microbubble synthesis. **Ana Torres:** Investigation, Formal analysis, Data curation. **Anxo Vidal:** Investigation, Formal analysis, Data curation. **Lars Dähne:** Writing – original draft, Supervision, Methodology, Investigation. **Daniel Razansky:** Writing – review & editing, Supervision, Resources, Methodology. **Xosé Luís Deán-Ben:** Writing – original draft, Visualization, Supervision, Resources, Project administration.

## Declaration of competing interest

The authors declare that they have no known competing financial interests or personal relationships that could have appeared to influence the work reported in this paper.

## Data Availability

Data will be made available on request.
