# Supplementary material for: Multiparametric super-resolution optoacoustic imaging with ICG-tagged microbubbles
Source: Mater Today Bio. 2025 May 12;32:101865. doi: 10.1016/j.mtbio.2025.101865 (PMC12148674; doi:10.1016/j.mtbio.2025.101865)
Supplement: Multimedia component 1 [file mmc1.docx]

**Supplementary Information**

**Multiparametric super-resolution optoacoustic imaging with ICG-tagged microbubbles**

Daniil Nozdriukhin^1,2^, Eva Remlova^1,2,3^, Lin Tang^1,2^, Shuxin Lyu^1,2,4^, Gabriella Egri^5^, Ana Torres^6^, Anxo Vidal^7^, Lars Dähne^5^, Daniel Razansky^1,2^, X. Luís Deán-Ben^1,2^

^1^Institute for Biomedical Engineering and Institute of Pharmacology and Toxicology, Faculty of Medicine, University of Zürich, Switzerland

^2^Institute for Biomedical Engineering, Department of Information Technology and Electrical Engineering, ETH Zürich, Switzerland

^3^Departement of Information Technology and Electrical Engineering, ETH Zürich and Max Planck ETH Center for Learning Systems, Switzerland

^4^Department of Medical Imaging, Shanxi Medical University, China

^5^Surflay Nanotec GmbH, Germany

^6^Experimental Biomedicine Centre (CEBEGA), University of Santiago de Compostela, Spain

^7^Center for Research in Molecular Medicine and Chronic Diseases (CiMUS), Health Research Institute of Santiago de Compostela (IDIS), University of Santiago de Compostela, Santiago de Compostela, Spain

**
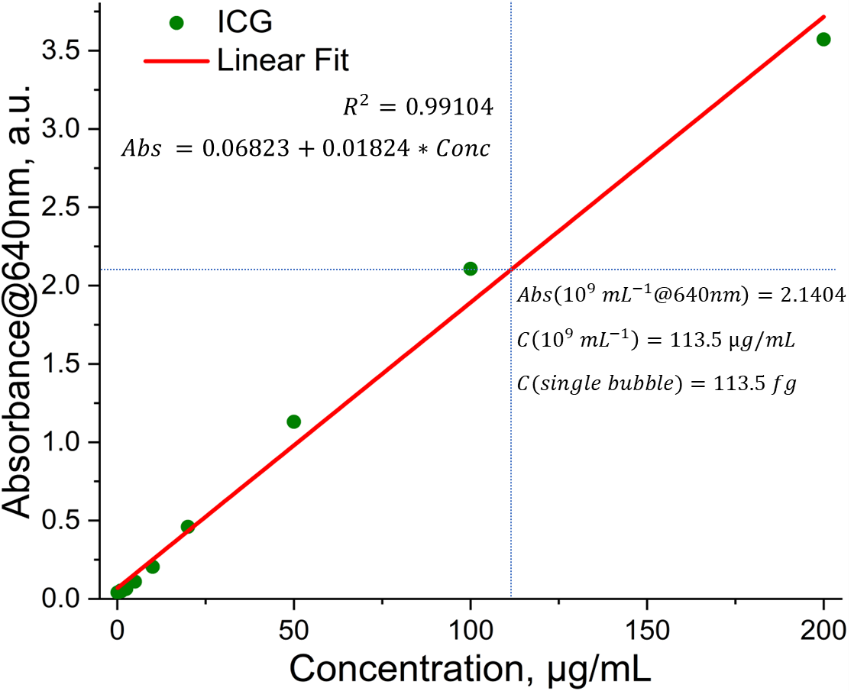
**

**Figure S1.** ICG per bubble estimation.

**Table S1:** Murine blood biochemistry data.

**Table S2:** Murine blood hematology data.

**
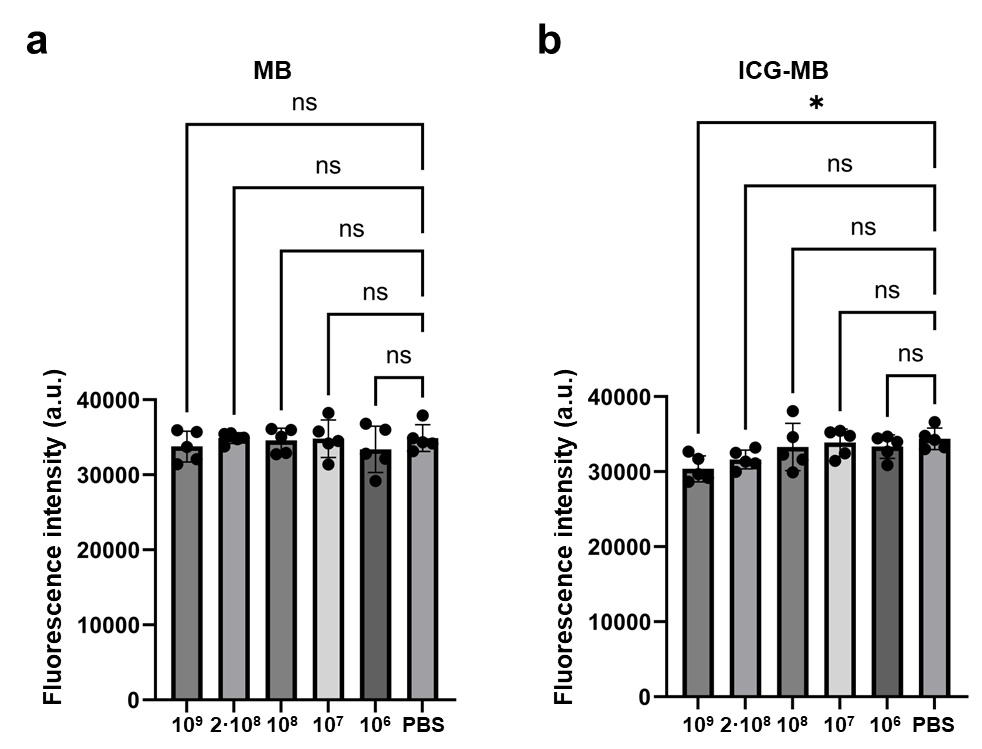
**

**Figure S2.** Statistical comparison of the cell viability data using Kruskal-Wallis test with Dunn multiple comparison correction of a) pristine microbubbles and b) ICG-labeled microbubbles; ns – non-significant, * - p < 0.05. The tests were performed in GraphPad Prism 10.3.0.


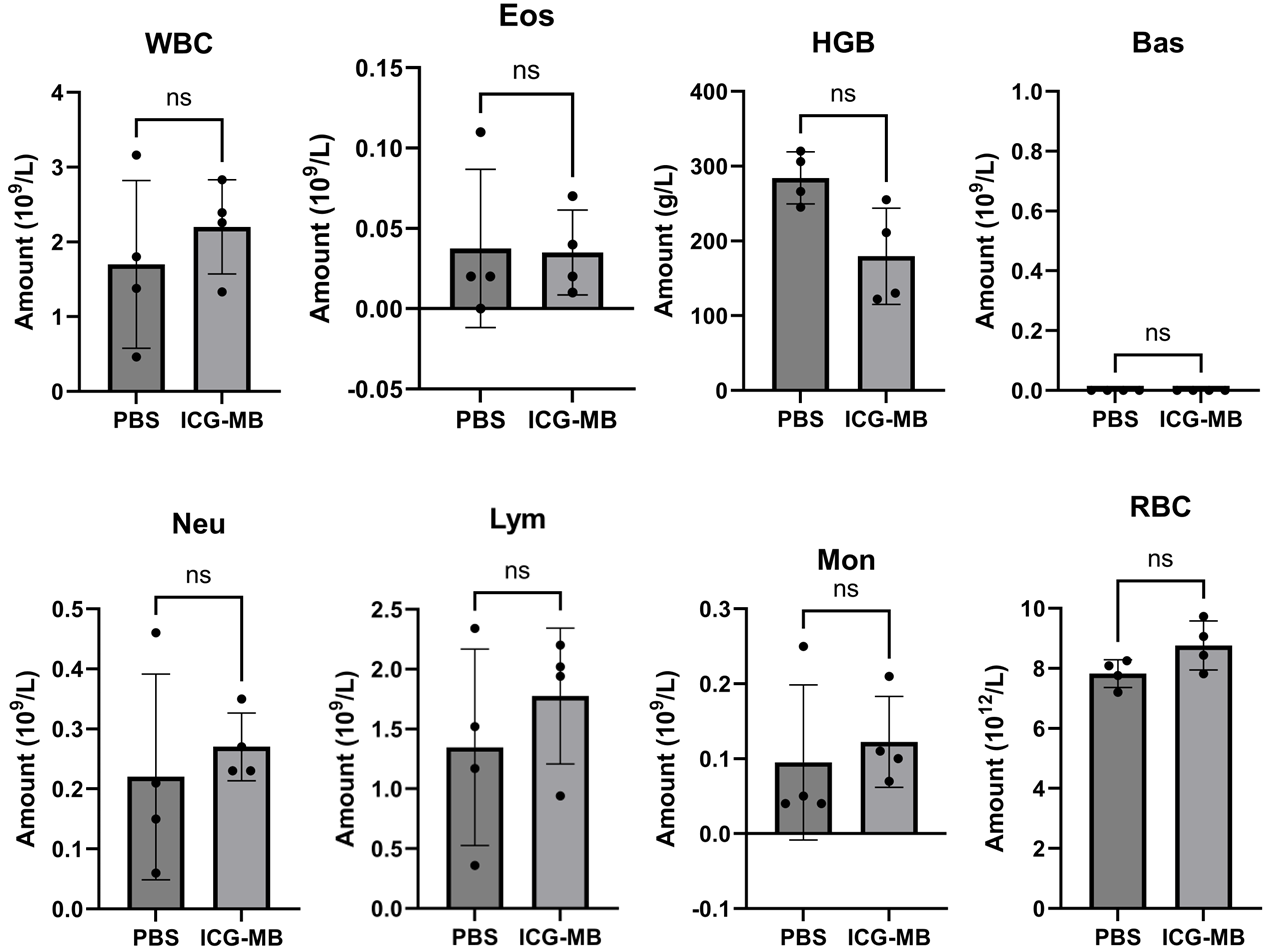


**Figure S3.** Statistical comparison of the murine blood hematology data using Mann-Whitney test between control group (PBS) and ICG-MB-injected group (ICG-MB); ns – non-significant, * - p < 0.05. The tests were performed in GraphPad Prism 10.3.0.

**
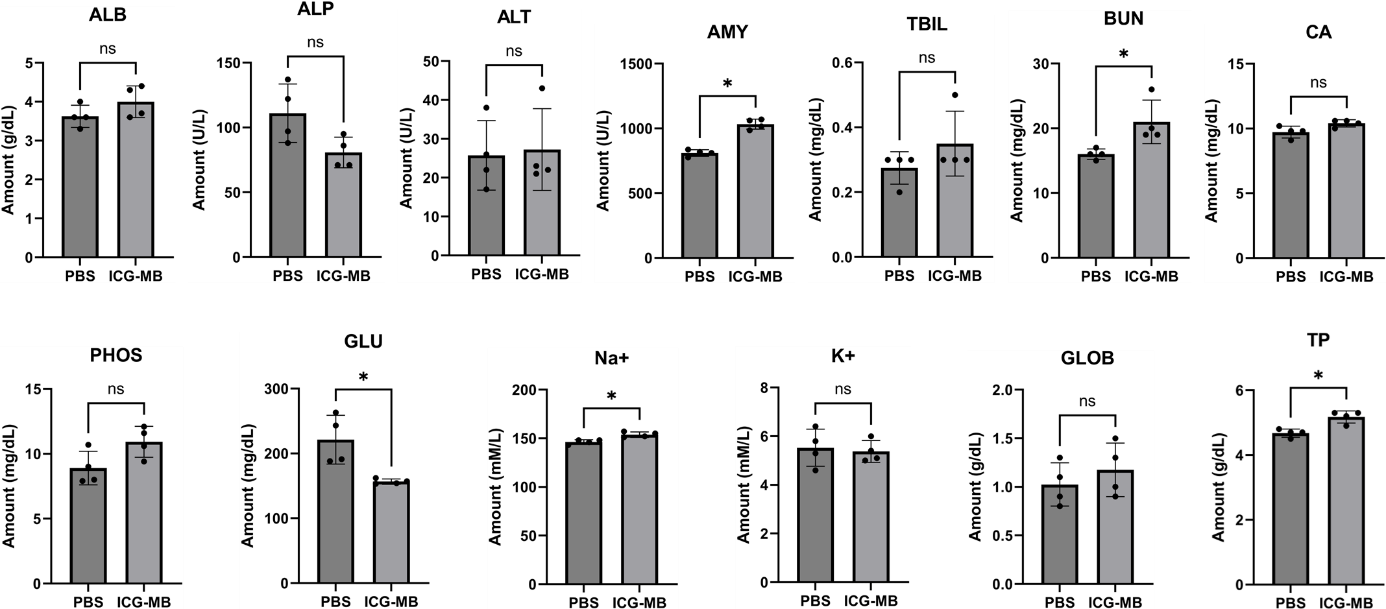
**

**Figure S4.** Statistical comparison of the murine blood biochemistry data using Mann-Whitney test between control group (PBS) and ICG-MB-injected group (ICG-MB); ns – non-significant, * - p < 0.05. The tests were performed in GraphPad Prism 10.3.0.


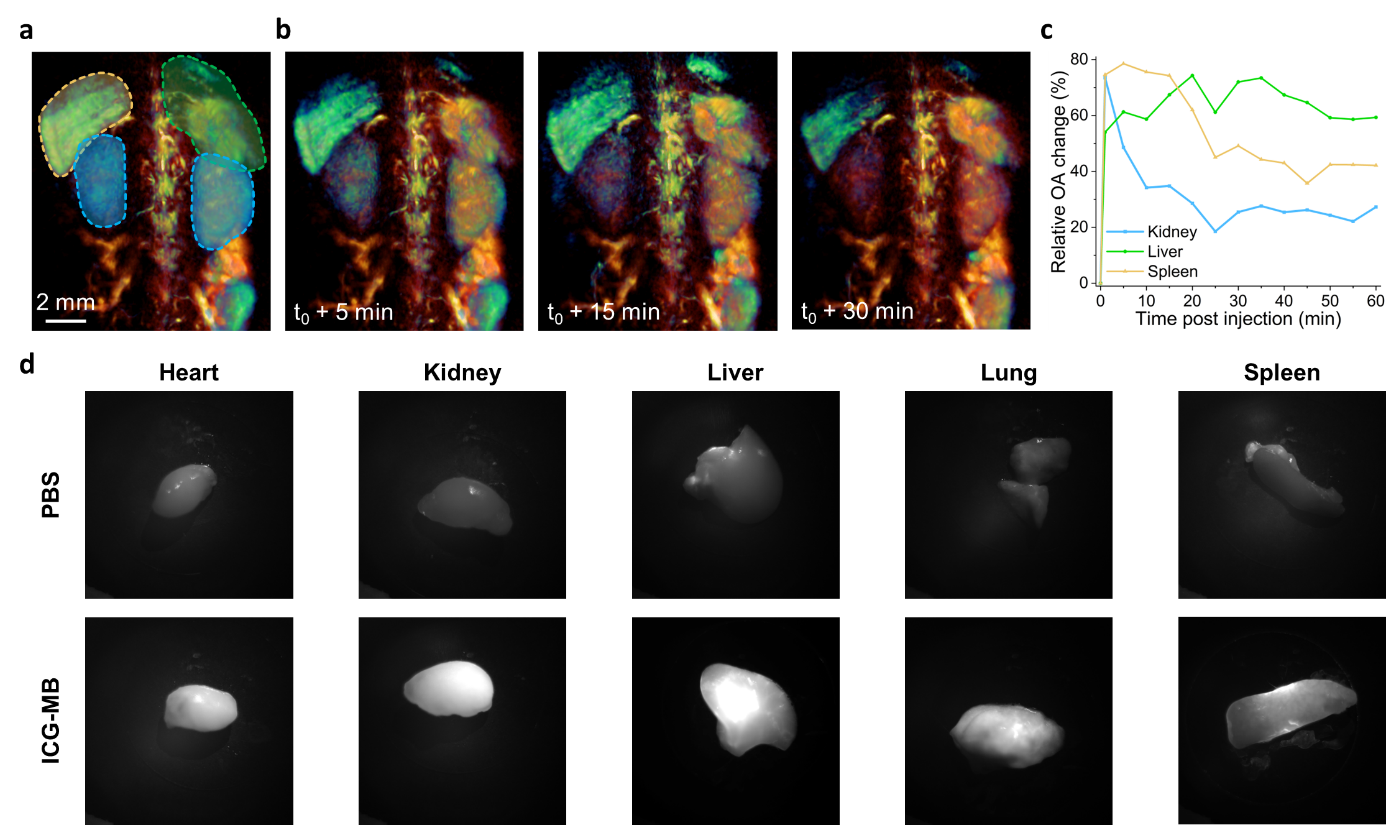


**Figure S5.** a) Single-sweep volumetric optoacoustic tomography (sSVOT) image of the murine back with major organs highlighted – spleen (yellow), liver (green) and kidneys (blue); b) Evolution of the OA signal with time post-injection, ICG-MB signal (green) is overlaid on the blood signal reference (orange); c) Accumulation curves extracted from the organs; d) Fluorescence images of the post-mortem extracted organs of the control mouse, injected with PBS and the mouse, injected with ICG-MB 60 min post-injection.

**
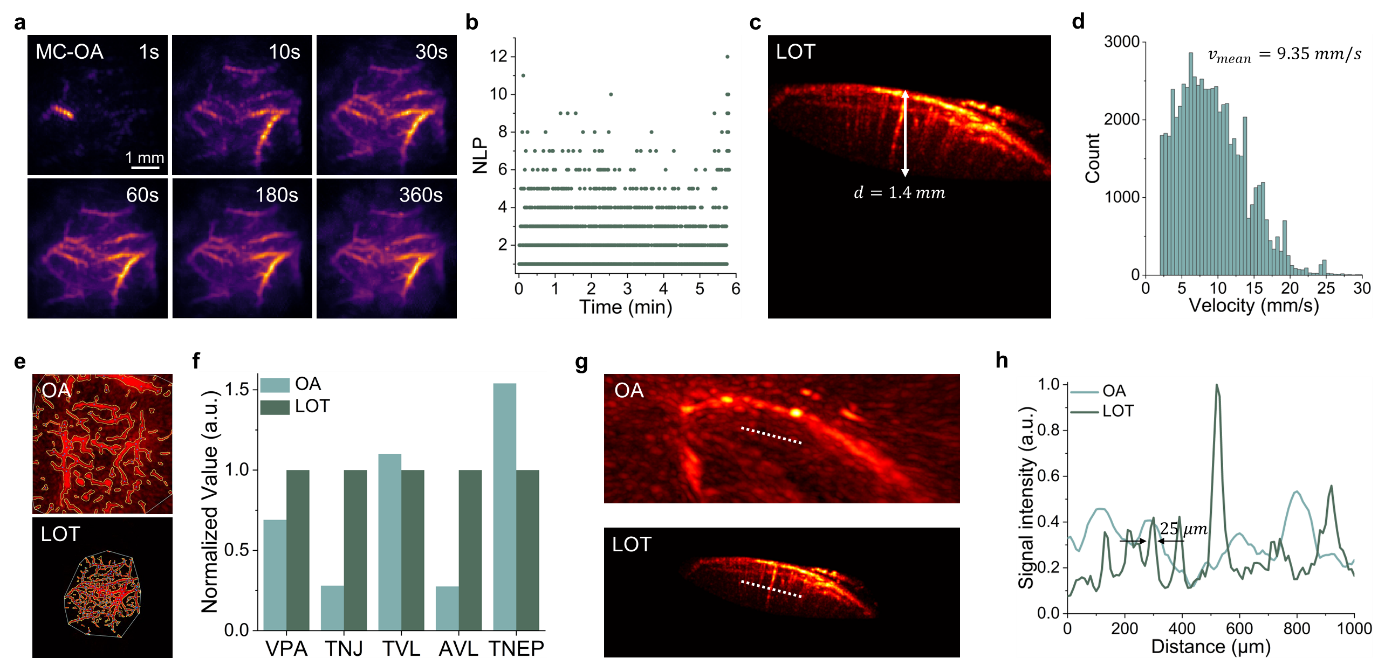
**

**Figure S6.** a) Dependence of the image quality on the acquisition time, motion-contrast optoacoustic images (MC-OA); b) the dependence of number of localized points per frame (NLP) on the time post-injection; c) the achieved depth of the LOT imaging with the ICG-MB; d) Velocity histogram of the vasculature; e) AngioTool 0.6a results of the OA and LOT image processing; f) Comparison of OA and LOT in vasculature detection ability: vessel percentage area (VPA), total number of junctions (TNJ), total vessel length (TVL), Average Vessels Length (AVL) and total number of endpoints (TNEP) are indicated; g) Vertical MIPS and corresponding profiles (white dotted lines) used for resolution comparison; h) Profiles along the white dotted line from panel (g).

**Video S1.** OA imaging of the ICG-MB flow in the 500 µm inner diameter polyethylene tubing at 10^6^ mL^-1^ concentration.

**Video S2.** OA and SVD-filtered videos of the ICG-MB, circulating in the mouse brain vasculature.

**Video S3.** Rotating views of the LOT, Velocity Map and oxygenation data, overlaid on LOT.
